# EXPLORING THE IMPACT OF INTERMITTENT HYPOXIA THROUGH A COMPREHENSIVE MULTI-OMICS APPROACH IN AGED MOUSE MODELS

**DOI:** 10.1093/geroni/igae098.2625

**Published:** 2024-12-31

**Authors:** Stefano Donega, Ross McDevitt, Rolando Hernandez, Cindy Chisolm, Kenneth FIshbein, Elizabeth McCart, Brian Wilgenburg, Luigi Ferrucci

**Affiliations:** National Institute on Aging, Baltimore, Maryland, United States; National Institute on Aging, Baltimore, Maryland, United States; National Institute on Aging, Baltimore, Maryland, United States; National Institute on Aging, Baltimore, Maryland, United States; National Institute on Aging, Baltimore, Maryland, United States; National Institute on Aging, Baltimore, Maryland, United States; National Institute on Aging, Baltimore, Maryland, United States; National Institute on Aging, Baltimore, Maryland, United States

## Abstract

Comprehending aging’s molecular mechanisms, like RNA splicing and AMPK signaling, is vital for tackling age-related issues and boosting resilience. Oxygen fluctuations from obstructive sleep apnea (OSA) cause Intermittent Hypoxia (IH), affecting respiratory functions and health, prompting adaptive responses to counter reduced energy production. Mice as models allow simulating disorders and studying muscle cell protection under low oxygen levels. Over the past two years, we have collected more than 1500 samples from 15 different tissues of adult (9-11 months) and old (20-23 months) mice exposed to IH at 21% to 5% oxygen concentration in 2:30 minutes cycles, for 8 hours a day over 4-8 weeks, aiming to construct a comprehensive murine IH atlas by employing cutting-edge technologies across diverse tissues. Analyses include conducting detailed transcriptomic analyses of muscle tissues using hybrid RNA-seq to identify changes in splicing isoforms during IH experiments, exploring transitions between fast-to-slow fiber types, studying mitochondrial morphology using C57/BL6 and mtKeima mouse strains (both wild-type and Alzheimer’s disease models), investigating brain spatial transcriptomics to understand the neurological effects of IH, analyzing cardiorespiratory disruptions by examining the epigenetic clock of the heart and spleen, and delving into the role of liver extracellular vesicles (EVs), particularly exosomes, in our sensitive mouse IH model. The overarching goal of this project is to integrate omics data comprehensively to elucidate the impact of alternate hypoxia on health and aging and develop innovative therapeutic strategies that minimize damage in patients affected by this highly prevalent conditions in older persons.

